# Cadherin 23-C Regulates Microtubule Networks by Modifying CAMSAP3’s Function

**DOI:** 10.1038/srep28706

**Published:** 2016-06-28

**Authors:** Satoe Takahashi, Vincent J. Mui, Samuel K. Rosenberg, Kazuaki Homma, Mary Ann Cheatham, Jing Zheng

**Affiliations:** 1 Department of Otolaryngology – Head and Neck Surgery, Feinberg School of Medicine, Northwestern University, Chicago IL 60611, USA; 2 Department of Communication Sciences and Disorders, Northwestern University, Evanston, IL 60208, USA; 3 Knowles Hearing Center, Northwestern University, Evanston, IL 60208, USA

## Abstract

Cadherin-related 23 (CDH23) is an adhesive protein important for hearing and vision, while CAMSAP3/Marshalin is a microtubule (MT) minus-end binding protein that regulates MT networks. Although both CDH23 and CAMSAP3/Marshalin are expressed in the organ of Corti, and carry several protein-protein interaction domains, no functional connection between these two proteins has been proposed. In this report, we demonstrate that the C isoform of CDH23 (CDH23-C) directly binds to CAMSAP3/Marshalin and modifies its function by inhibiting CAMSAP3/Marshalin-induced bundle formation, a process that requires a tubulin-binding domain called CKK. We further identified a conserved N-terminal region of CDH23-C that binds to the CKK domain. This CKK binding motif (CBM) is adjacent to the domain that interacts with harmonin, a binding partner of CDH23 implicated in deafness. Because the human Usher Syndrome 1D-associated mutation, CDH23 R3175H, maps to the CBM, we created a matched mutation in mouse CDH23-C at R55H. Both *in vivo* and *in vitro* assays decreased the ability of CDH23-C to interact with CAMSAP3/Marshalin, indicating that the interaction between CDH23 and CAMSAP3/Marshalin plays a vital role in hearing and vision. Together, our data suggest that CDH23-C is a CAMSAP3/Marshalin-binding protein that can modify MT networks indirectly through its interaction with CAMSAP3/Marshalin.

CDH23, an atypical cadherin protein, belongs to the cadherin superfamily that plays a major role in cell adhesion. *CDH23* is widely expressed in various tissues including brain, heart, lung, kidney, nose, eye, and ear^1^,^2^ as well as in certain cancer cells^3^. Most of our knowledge regarding the functions of CDH23, however, comes from studies on hearing and vision, as mutation of *CDH23* is known to cause various degrees of hearing impairment and blindness, including Usher Syndrome 1D^4^, age-related hearing loss, and nonsyndromic deafness (autosomal recessive 12, DFNB12)^5^. In addition to its roles in development and cell differentiation^6^,^7^, the adhesive property of CDH23 permits formation of the tip link (in conjunction with PCDH15), which connects the stereocilia and kinocilia of inner ear hair cells^8^. Such sophisticated and delicate structures are crucial for transforming mechanical stimulation to electrical signals in the process of sensory hair cell transduction so that auditory information can be conveyed to the brain^9^.

There are three classes of *CDH23* isoforms (Fig. 1A) that are expressed at different times within different tissues^10^. Each isoform has two subtypes, which differ in their cytoplasmic domains such that subtype 1 contains exon 68 (encodes 35 amino acids), while subtype 2 does not. The extracellular portion of CDH23, which includes the EC repeats, and the transmembrane domains are only found in the A (associated with the tip link) and B isoforms. All isoforms have almost identical cytoplasmic domains except for the first 7 amino acids (aa) at the N-terminus of the C-isoform. Unlike isoforms A and B that function as adhesive proteins, very little is known about the role of CDH23-C, which is entirely cytoplasmic and has no direct adhesive function. Interestingly, CDH23 proteins were found around microtubule (MT)-rich areas when using antibodies that do not distinguish between the three isoforms, including the centrosome and basal bodies in adult mouse cochleae and the synaptic terminals of both hair cells, photoreceptor cells and afferent spiral ganglion neurons^10^,^11^,^12^,^13^. Although the physiological role(s) of CDH23 in these MT-rich areas is unknown, CDH23-C may be the predominant isoform to localize to these locations, as CDH23-C is not tethered to the membrane. In addition, all identified CDH23 intracellular partners, such as membrane-associated guanylate kinase, WW and PDZ domain-containing protein 1 (MAGI-1)^14^, EHD4^15^, cyclic nucleotide-gated channel α-3 (CNGA3)^16^ and harmonin^17^,^18^,^19^ are not MT-associated. These observations raise the possibility that CDH23, particularly CDH23-C isoform, may have distinct partners that link CDH23 to MTs.

Microtubules are an integral part of dynamic cytoskeletal networks that constantly reshape themselves to support various cellular functions. MT networks are regulated by many factors, including what are referred to as tracking proteins. There are two types of tracking proteins, plus end (+TIPs) and minus end tracking proteins (−TIPs), each of which targets the corresponding end of the MT and assists in maintaining the dynamic nature of the cytoskeletal network. In contrast to +TIPs, for which many have been identified, only a few −TIPs have been reported. Previously, we identified Marshalin (KIAA1543), a MT minus-end binding protein, as a potential CDH23-associated protein through a membrane-based yeast two hybridization screening^20^. Since Marshalin^21^ is described under many aliases including CAMSAP3^22^, patronin^23^, and Nezha^24^, we use the designation CAMSAP3/Marshalin in this report. As a newly discovered MT-minus-end associated protein, CAMSAP3/Marshalin has been reported to play various regulatory roles including stabilizing MTs^23^,^25^, permitting MT minus-end polymerization^26^, maintaining adhesive junction stability through its connection with Pleckstrin Homology Domain Containing, Family A Member 7 (PLEKHA7)^24^, and regulating MT networks^27^,^28^, particularly in noncentrosomal MT dynamics including axon regeneration and planar cell polarity (PCP)^29^,^30^. CAMSAP3/Marshalin contains multiple protein interaction domains (see Fig. 1B) including a carboxy-terminal CKK domain (residues 1112–1241, also named DUF1781) that recognizes and binds to the minus-end of MTs^22^, three predicted coiled-coil (CC) domains likely responsible for thick MT bundle formation^21^, an MT binding domain (MTB, designated as M in Fig. 1B) present in the linker region between CC2 and CC3 that is responsible for MT stabilization (Residues 727–840)^26^,^31^, and a predicted helical domain (H) immediately following the CC3 domain, which has also been reported to stabilize MTs^26^. A calponin homology (CH) domain is also located near the N-terminal of the protein. At the present time, it is not clear whether or how CAMSAP3/Marshalin interacts with other proteins through these protein-protein interaction domains.

In this study we investigated the relationship between CAMSAP3/Marshalin and CDH23-C proteins, as the two proteins are co-expressed in many cells including cochlear hair cells. Since both CDH23 and CAMSAP3/Marshalin carry several protein-protein interaction domains, we systemically characterized the authenticity of interactions between CAMSAP3/Marshalin and CDH23-C using both *in vitro* pull-down assays and *in vivo* CAMSAP3/MT bundle formation methodologies. We demonstrate that the CKK domain of CAMSAP3/Marshalin binds to the N-terminus of CDH23-C, which we named the CKK binding motif (CBM). Interestingly, a missense mutation has been found within the CBM of CDH23 (R3175H) in Usher Syndrome 1D patients^32^. We, therefore, investigated the functional consequences of this mutation and discovered that R3175H negatively affects CKK binding, suggesting a possible impairment in MT regulation that may contribute to the clinical manifestations. Taken together, our data link the CDH23 cytoplasmic regions to MT networks via CAMSAP3/Marshalin, and suggest a novel role for CDH23-C in regulating MT networks.

## Results

### CDH23-C inhibits CAMSAP3/Marshalin-induced MT bundle formation

Previously, we showed that overexpression of CAMSAP3/Marshalin-Ld (“L” stands for long isoform; “d” stands for subtype d) resulted in dramatic reorganization of MT networks into bundles composed of both CAMSAP3/Marshalin and tubulin^21^. Consistent with Jiang and colleagues’ report, we observed co-localization of CAMSAP3/Marshalin throughout the entire length of MT bundles, not just at their minus-ends (Fig. 2A–C)^26^. Treatment of OK cells with colchicine, a MT polymerization inhibitor, prevented formation of MT bundles induced by CAMSAP3/Marshalin, indicating that this process requires MT polymerization (Fig. 2D–F). This MT bundle formation is observed in cells overexpressing CAMSAP3/Marshalin, but not in cells overexpressing CDH23-C isoforms, as the OK cells overexpressing CDH23-C isoforms have typical MT networks, which are indistinguishable from nearby non-transfected cells (Supplementary Figure S1). This result indicates that CDH23-C proteins alone do not affect MT networks in host cells, where native CAMSAP3/Marshalin-Ld expression is low. This result confirms the specificity CAMSAP3/Marshalin and tubulin interaction for the re-organization of MT formations. Since both CDH23-C1 and C2 constructs were tagged with GFP at the C-terminus, we also verified that GFP alone did not change MT networks or interfere with CAMSAP3/Marshalin-induced bundle formation (Fig. 2G–J). Although not shown here, transient transfection of OK cells with Ceacam16, an adhesive protein expressed within the organ of Corti^33^, also did not induce MT bundles. In contrast, co-expression of CDH23-C1 with CAMSAP3/Marshalin in OK cells dramatically reduced the formation of CAMSAP3/Marshalin-induced MT bundles (Fig. 2K–N).

To further evaluate the interaction between CDH23-C and CAMSAP3/Marshalin, we classified CAMSAP3/Marshalin expression patterns in cells that co-expressed CDH23-C isoforms (Fig. 3A), as in our previous study^21^. Type I signifies “speckles” distributed throughout the cytoplasm; type II short “sticks” and type III long “strings”. When CAMSAP3/Marshalin is co-expressed with GFP in OK cells, more than half of the cells showed type III patterns (54.1%), while 20.8% were type I and 25.1% were type II (Fig. 3B). Cells with type III patterns were significantly decreased when CAMSAP3/Marshalin-Ld was co-expressed with CDH23-C1-GFP (*p* = 0.0013) and CDH23-C2-GFP (*p* = 0.0037): by 21.3% for CDH23-C1 and by 20% for CDH23-C2, respectively. Thus, type I cells dominated when OK cells co-expressed CAMSAP3/Marshalin-Ld and CDH23-C isoforms. In contrast, a majority of cells co-expressing CAMSAP3/Marshalin-Ld and Ceacam16 showed type III MT patterns similar to the expression patterns of cells expressing CAMSAP3/Marshalin-Ld and GFP (Supplementary Figure S2). This result indicates that Ceacam16 did not interfere with the formation of thick bundles induced by CAMSAP3/Marshalin-Ld expression. We also co-expressed CAMSAP3/Marshalin-Ld and CDH23-C1 in LLC-PK1-Cl4 cells^19^,^34^. The ability of CDH23-C isoforms to interfere with CAMSAP3/Marshalin-induced MT bundle formation was also observed (Supplementary Figure S3) in this epithelial cell line. In summary, CDH23-C isoforms inhibit CAMSAP3/Marshalin-induced MT bundle formation independent of host cell identity.

### Identification of the binding sites between CAMSAP3/Marshalin-Ld and CDH23

**Figure 1:**
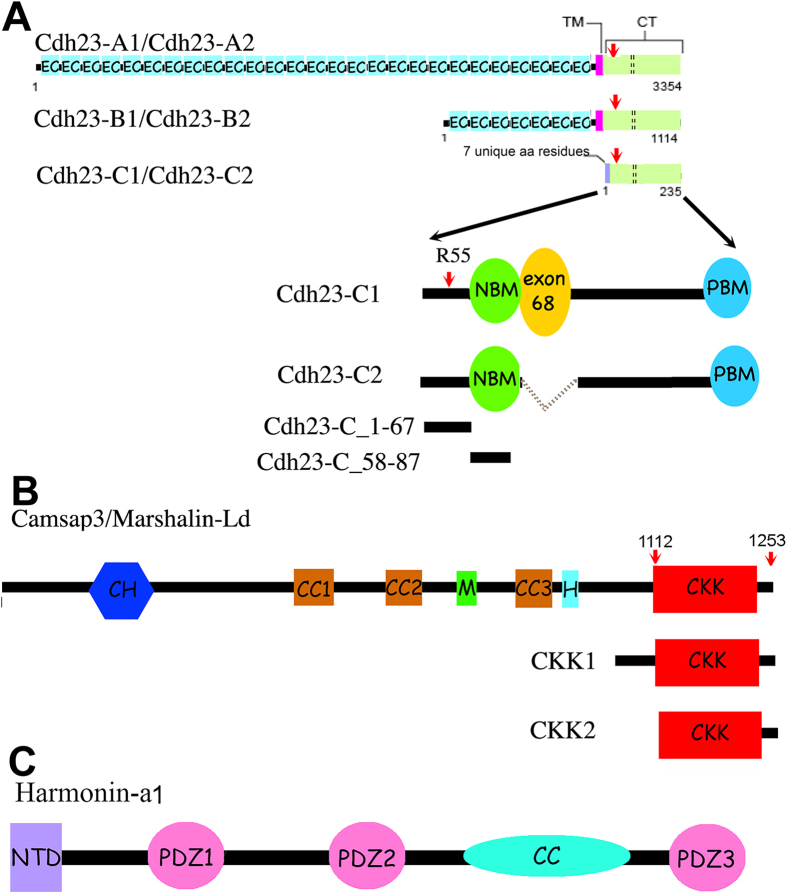
A schematic representation of domain organization and corresponding cDNA constructs of mouse CDH23 isoforms, CAMSAP3/Marshalin-Ld, and harmonin-a1. (Panel **A**) Three CDH23 isoforms and their subtypes. *Cdh23* undergoes alternative splicing that involves the inclusion (subtype 1) or exclusion (subtype 2) of exon 68 (shown as an orange circle in CDH23-C1 or as dotted lines for CDH23-C2), which encodes 35 cytoplasmic amino acids. CDH23-A consists of 27 extracellular cadherin (EC) repeats, a transmembrane (TM) domain (pink box), and a cytoplasmic domain (CT, pale green box), which contains PDZ-binding Motifs (PBM), and the N-terminal domain of a harmonin binding motif (NBM) (details shown in CDH23-C1). The B isoform proteins differ from CDH23-A in that they have seven EC motifs. The C isoforms are cytosolic and do not have EC repeats or the TM. C1 and C2 both have seven novel amino acids coded by exonic sequences in the N-terminus. The red arrow indicates the R3175H position in the CT, and the corresponding mutation at R55 in mouse *Cdh23-C*. (Panel **B**) CAMSAP3/Marshalin-Ld and the CKK domain constructs. CAMSAP3, also called Marshalin-Ld^21^, codes three CC (coiled-coil) domains (brown), CKK (a carboxy-terminal tubulin-binding) domain (red), the CH (calponin homology) domain (blue), a microtubule-binding (M) domain (green), and a helical (H) domain (light blue). Although CKK1 (resides 1020–1253) and CKK2 (resides 1112–1253) both contain the CKK domain, CKK1 includes an extra region N-terminus to the CKK domain, and derived from CAMSAP3/Marshalin-Ld. (Panel **C**) Harmonin-a1. Harmonin-a1 contains the N-terminal domain (NTD) (purple), three PDZ (PSD-95/Dlg/ZO-1) domains (pink), and a predicted CC domain (turquoise).

**Figure 2:**
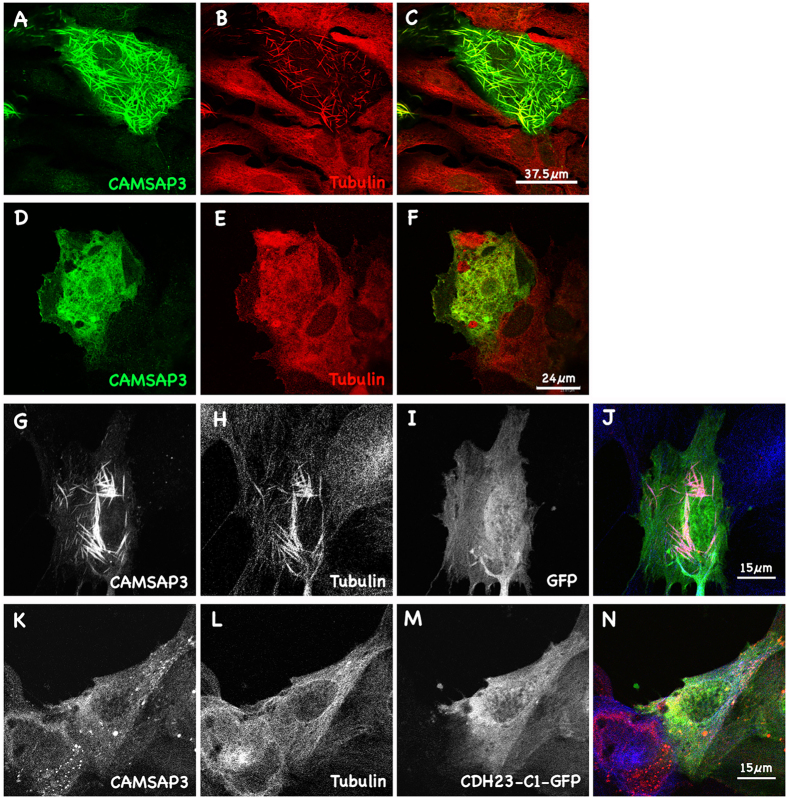
CAMSAP3/Marshalin re-organizes cytoskeletal networks. (**A–C)** Immunofluorescent images showing MT bundles induced by CAMSAP3/Marshalin-Ld. A merged image (**C**) shows both CAMSAP3/Marshalin-Ld (**A**) and MT staining (**B**). (**D–F)** CAMSAP3/Marshalin-Ld-induced MT bundles disappeared when cells were treated with 43 nM colchicine. A merged image (**F**) shows CAMSAP3/Marshalin-Ld (**D**) and tubulin (**E**) staining. (**G–J**) Co-transfection of GFP with CAMSAP3/Marshalin-Ld does not interfere with thick bundle formation. A merged image (**J**) shows CAMSAP3/Marshalin-Ld (**G**), Tubulin (**H**), and GFP (**I**). **(K–N)** MT bundles disappeared when cells were co-transfected with CAMSAP3/Marshalin-Ld and CDH23-C1-GFP. A merged image (**N**) shows CAMSAP3/Marshalin-Ld (**K**), Tubulin (**L**), and CDH23-C1-GFP (**M**). CAMSAP3/Marshalin-Ld was stained using anti-Marshalin (**A,D,G,K**); MTs by anti-α-tubulin (**B,E,H,L**).

**Figure 3:**
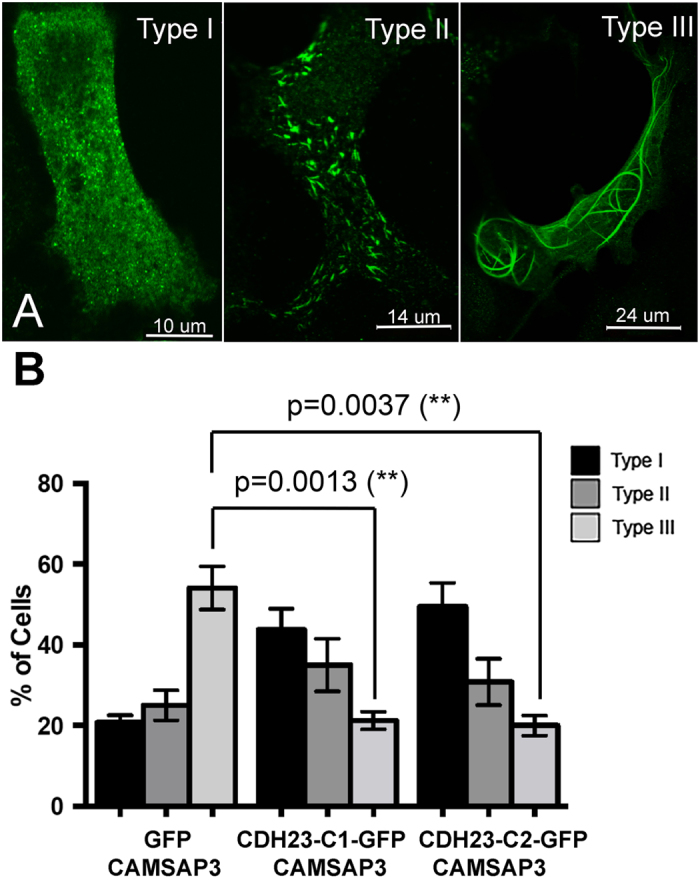
CDH23-C inhibits MT bundle formation. (**A)** Immunofluorescent images show three different expression patterns of CAMSAP3/Marshalin-Ld. CAMSAP3/Marshalin-Ld was detected using an anti-V5 antibody followed by goat anti-mouse IgG-Alexa Fluor 488 antibody. **(B)** A bar graph showing the distribution of three expression patterns of CAMSAP3/Marshalin-Ld in OK cells 24 hours after co-transfection of *CAMSAP3/Marshalin-Ld* with *GFP, CDH23-C1-GFP*, and *CDH23-C2-GFP,* respectively. Type III bundles are statistically decreased in the presence of CDH23-C1 (n = 4, *p = *0.0013) and CDH23-C2 (n = 3, *p = *0.0037) as compared to GFP (n = 4). Means ± s.e.m. are shown.

**Figure 4:**
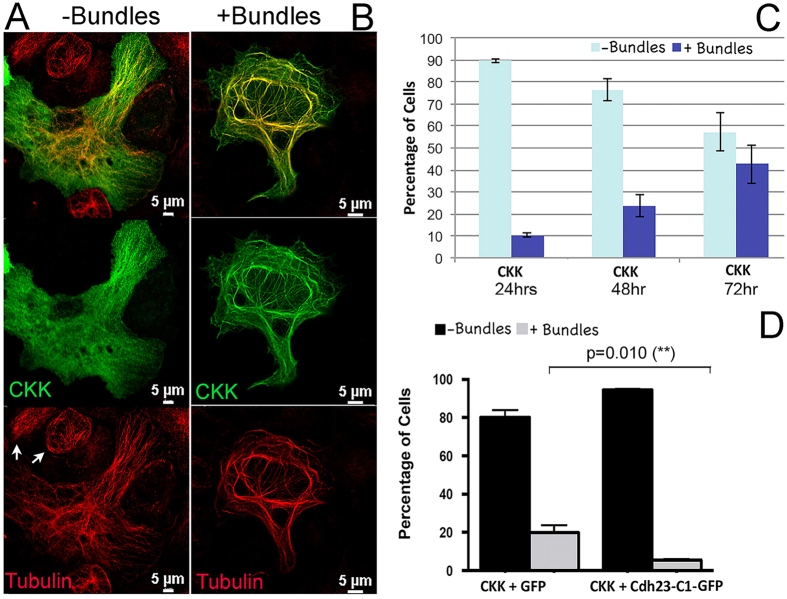
The CKK domain of CAMSAP3/Marshalin-Ld induces MT bundle formation. (**A**,**B**) Immunofluorescent images show two different expression patterns of CKK fragments. CKK was detected by anti-Marshalin followed by goat anti-rabbit IgG-Alexa Fluor 488 antibodies. MTs were detected by anti-α-tubulin followed by goat anti-mouse IgG-Alexa Fluor 546 antibodies. (**C**) A bar graph shows the distribution of two expression patterns of CKK in OK cells 24, 48 and 72 hours post transfection with plasmids encoding CKK1-V5-His. (**D**) A bar graph shows the distribution of two expression patterns of CKK in OK cells 48 hours post transfection with plasmids encoding *CKK1 + GFP* or *CKK1 + CDH23-C1-GFP*, respectively. Cells with bundles are statistically decreased in the presence of CDH23-C1 (n = 4, *p = *0.010) as compared to GFP. Means ± s.e.m. is shown.

To understand the molecular mechanisms underlying the inhibitory role of CDH23-C isoforms on CAMSAP3/Marshalin-induced MT bundle formation, we first determined the functional domains that are responsible for the CDH23-C and CAMSAP3/Marshalin interaction. CAMSAP3/Marshalin-Ld was identified as a potential CDH23-associated protein using the membrane-based yeast two-hybridization (Y2H) approach^20^. Sequence analyses suggested that CDH23 was associated with the CKK domain of CAMSAP3/Marshalin. Since the CKK domain is known to directly interact with tubulin^22^, we tested whether the CKK domain of CAMSAP3/Marshalin alone could induce bundle formation similar to full-length CAMSAP3/Marshalin-Ld. In these experiments, OK cells were transiently transfected with a plasmid encoding CAMSAP3/Marshalin-Ld’s CKK domain (CKK1, residues 1020–1253) (Fig. 1B). Similar to the full-length CAMSAP3/Marshalin-Ld, CKK1-induced bundles co-stained with both anti-Marshalin and anti-tubulin antibodies (Fig. 4B), suggesting that these bundles are composed of both CKK and tubulin. Similar to full-length CAMSAP3/Marshalin-Ld, CKK1-induced MT bundle formation also requires MT polymerization, as addition of colchicine abolished all CKK1-induced MT bundles in CKK1-expressing cells. However, the robustness of CKK1-induced MT bundles and the percentage of CKK1-expressing cells displaying such bundles were significantly less than in cells that were transfected by full-length CAMSAP3/Marshalin-Ld (Fig. 3). This reduction may relate to the lack of MT stabilizing elements, the MTB, and/or other helical structures in the CKK1 construct (Fig. 1B). Therefore, we categorized these CKK1-expressing cells into those without CKK bundles (−bundles) or those with (+bundles) as shown in Fig. 4A,B. Cells lacking CKK bundles display normal MT networks (red staining), similar to nearby non-transfected OK cells (Fig. 4A, see arrows in the “Tubulin” image). In these cells, CKK is distributed throughout the cytoplasm without obvious bundle staining but with few “speckles” overlaping tubulin staining (Fig. 4A, see “CKK”). In contrast, the cell with CKK1-induced MT bundles has long, curved bundles composed of both tubulin (red) and CKK proteins (green) as shown in Fig. 4B. The number of cells with CKK-induced MT bundles increased as more CKK was expressed, as shown in Fig. 4C. Only 9% of CKK1-expressing cells showed CKK1-induced MT bundles at 24 hours post CKK1 transfection, while the numbers of cells with MT bundles increased to 44% after 72 hours (Fig. 4C). Thus, the degree of CKK1-induced MT bundle formation seems to correlate with the amount of CKK1 protein produced. Such time-dependent bundle formation was also observed in OK cells transfected with full-length CAMSAP3/Marshalin-Ld^21^. To verify whether CDH23-C isoforms also interfere with MT bundle formation induced by CKK1, OK cells were co-transfected with plasmids encoding CKK1 and the CDH23-C1 isoform or GFP. 44 to 68 hours following transient transfection, 19.9% of CKK1/GFP-expressing cells showed CKK1-induced MT bundles, compared to only 5.5% of CKK1/CDH23-C1-expressing cells (Fig. 4D, n = 4, *p* = 0.010). Thus, CDH23-C1-GFP co-expression interferes with CKK’s ability to induce MT bundles, similar to that seen for full-length CAMSAP3/Marshalin-Ld.

**Figure 5:**
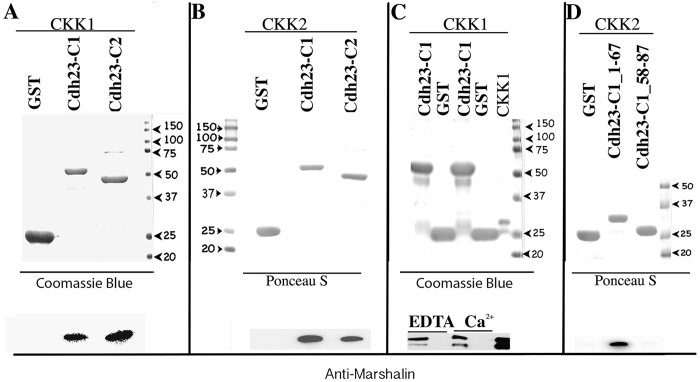
CKK directly binds to the N-terminus of CDH23-C isoforms in a Ca^2 + ^-insensitive fashion. (**A**) GST pull-down of CDH23-C1 and C2 with CAMSAP3/Marshalin-Ld’s CKK domain. **Top:** GST, GST-tagged CDH23-C1 and CDH23-C2 proteins are immobilized on glutathione-agarose, and incubated with cell lysates containing CKK1 proteins. GST-fusion proteins were visualized by Coomassie Blue. **Bottom**: Both CDH23-C1 and CDH23-C2 pulled down CKK1, while GST alone did not. CKK1 was identified using anti-Marshalin. (**B)** GST pull-down assay of CKK2 and CDH23-C isoforms, showing binding between CKK2 and CDH23-C1 or CDH23-C2, respectively. (**C)** GST pull-down assay of purified CKK1 and Cdh23-C1, showing binding in the no Ca^2 + ^(1 mM EDTA) and 1 mM Ca^2 + ^conditions. (**D**) GST pull-down assay of CKK2 and CDH23-C1 fragments, showing the binding between CKK2 and the N-terminus containing aa 1–67 of CDH23-C1, but no binding between CKK2 and Cdh23-C1_58–87. As in (**A**) GST, GST-tagged CDH23-C1 and CDH23-C2 proteins were visualized by either Ponceau S staining or Coomassie Blue in upper panels, while CKK1 and CKK2 were identified by anti-Marshalin (bottom panels of (**B,C,D)**. Molecular weights (in kD) are labeled with numbers and arrowheads.

To assess if CKK and CDH23-C isoforms directly interact, we examined binding between CDH23-C isoforms and CKK domain using *in vitro* pull-down assays. GST-tagged CDH23-C1 and CDH23-C2 fusion proteins were expressed in bacteria and immobilized on glutathione-agarose. The glutathione resins with GST-alone or GST-CDH23-C isoforms were then incubated with bacterial lysates containing the CKK domain, and bound proteins were eluted and detected on SDS-PAGE. The presence of GST, and GST-CDH23-C isoforms were detected by Coomassie Blue staining (Fig. 5A, top), and the CKK domains were detected by anti-Marshalin (Fig. 5A, bottom). CKK1 (residues 1020–1253, see Fig. 1B) is pulled down by GST-CDH23-C1 and C2 but not by GST alone (Fig. 5A control lane). Similar to CKK1, CKK2 (residues 1112–1253), which only contained the predicted CKK domain, was also pulled down by both CDH23-C isoforms (detected by Ponceau S) (Fig. 5B). These data confirm the physical interaction between CKK and CDH23-C isoforms, and that exon 68 is not required. To exclude the possibility that bacterial proteins present in the crude lysate mediate the CKK and CDH23-C interaction, we also purified the CKK1 domain first using Ni-NTA beads before mixing with GST-CDH23-C-bound resin. Purified CKK1 bands were observed along with GST-CDH23-C1 (Fig. 5C, top). GST-CDH23-C1, but not GST alone, pulled down purified CKK, confirming direct binding (Fig. 5C, bottom). Since both MT polymerization and the adhesive function of CDH23 are regulated by calcium, we also tested the role of calcium in CDH23-C and CKK interaction. As shown in Fig. 5C (bottom), the interaction between CDH23-C1 and CKK1 is calcium-insensitive, as the presence of 1 mM EDTA did not affect the binding observed in the presence of 1 mM CaCl_2_ (Fig. 5C). Taken together, CDH23-C isoforms are capable of directly interacting with the CKK domain of CAMSAP3/Marshalin-Ld independent of calcium concentration and the presence of exon 68 in CDH23.

Cytoplasmic regions of CDH23 have been reported to interact with other proteins including harmonin through the PDZ domain-binding motif (PBM), exon 68, or the N-terminus domain binding motif (NBM) as shown in Fig. 1C^17^,^18^,^35^. To further map the region involved in CKK binding, we tested the interaction between CKK and the GST-tagged cytoplasmic tail of mouse CDH23 constructs derived from CDH23-A1 and A2 [19] by *in vitro* GST pull-down assays. Cytoplasmic tails from CDH23-A1 and A2 differ from the C isoforms by having an extra 62 amino acids ahead of the N-terminus of the C isoform and by lacking 7 residues unique to C isoforms. Deletions from the C-terminus, including deletion of the C-terminal PBM (Cdh23 (+68, Δ4)), exon 68 (Cdh23 (Δ108)), and NBM (Cdh23 (Δ143) (see Methods for details), did not abolish binding between CKK and CDH23 (Supplementary Figure S4). This result indicates that CKK-binding may be mediated by an N-terminal region upstream of the NBM of CDH23’s cytoplasmic domain, and does not involve previously characterized harmonin binding sites (PBM, exon 68, and NBM). To test this possibility, we constructed two plasmids, one encoding the first 67 residues (Cdh23-C_1–67) of the C-isoform and including the unique 7 amino acids, and the second encoding amino acids 58–87 (Cdh23-C_58–87) and containing the harmonin-binding domain NBM (Fig. 1C), to be used in the pull down assay. CKK2 fragments (smaller construct, see Fig. 1B) were mixed with GST or GST-tagged proteins immobilized on glutathione-agarose. GST and GST-tagged proteins were detected by Ponceau S (Fig. 5D, top), while CKK2 was detected using anti-Marshalin antibody (Fig. 5D, bottom). GST-CDH23-C_1–67 pulled down CKK2 while GST alone and GST-CDH23-C_58–87 did not, even though excess CKK2 fragments were found in the flow through. This result further confirms that the binding site for CKK is located at the beginning of the CDH23-C, but does not include or require the harmonin-binding site NBM. We named this region (residues 1–67) the CKK binding motif (CBM).

### CDH23-C is able to bind CAMSAP3/Marshalin and harmonin simultaneously

**Figure 6:**
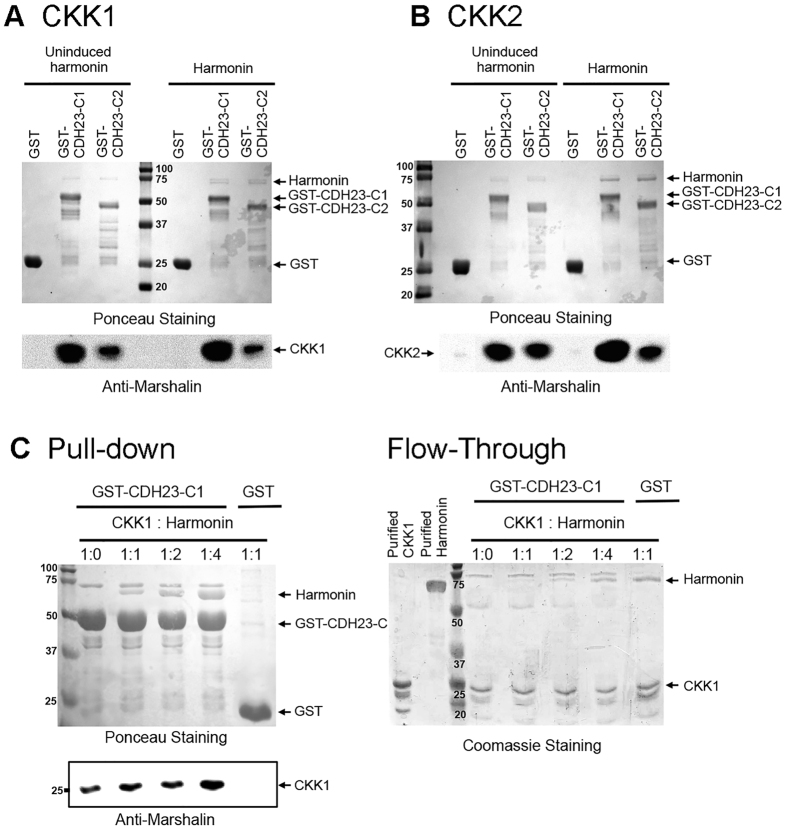
CDH23-C isoforms are able to bind CKK and harmonin simultaneously. GST, GST-tagged CDH23-C1 and CDH23-C2 proteins were immobilized on glutathione-agarose and incubated with equal amounts of cell lysates containing CKK domains with or without harmonin (uninduced). GST-agarose associated proteins were eluted with 100 mM L-glutathione and separated on a 12% SDS-PAGE gel. GST, GST-tagged proteins, and harmonin were visualized by Ponceau S at the top, while CKK1 (panel **A**) and CKK2 (panel **B**) were identified using anti-Marshalin (bottom). Both CDH23-C1 and CDH23-C2 pulled down CKK1 and 2 in the presence or absence of harmonin, while GST alone did not. In panel **(C**) CKK1 and harmonin were first purified separately. Similar amounts of CDH23-C1-GST bait and GST were mixed with 1 μM of CKK1 and harmonin at different molar concentrations, including CKK1: harmonin ratios of 1:0, 1:1, 1:2, and 1:4. Molecular weights (in kD) are labeled with numbers. The excess unbound CKK1 and harmonin were visualized by Coomassie Blue.

Our data demonstrated that CDH23-C binds to CAMSAP3/Marshalin’s CKK domain but the minimum binding region did not include one of the harmonin binding sites (NBM). To learn more about the relationship among CAMSAP3/Marshalin, harmonin, and CDH23-C isoforms, we tested whether CDH23-C isoforms could still bind to CAMSAP3/Marshalin in the presence of harmonin. GST-tagged CDH23-C1 and CDH23-C2 proteins were first immobilized on glutathione-agarose along with GST as a negative control. GST-agarose and CDH23-C-GST-agarose were then simultaneously incubated with lysates from bacteria expressing CKK and lysates from bacteria expressing harmonin. In paired comparisons, glutathione-agarose with GST or GST-CDH23-C isoforms were incubated with CKK lysates and uninduced harmonin bacterial lysates, which were derived from the same bacterial transformants without IPTG treatment. The latter serves as the negative control as it contains similar proteins except for harmonin. As shown in Fig. 6, GST-CDH23-C1 and C2 pulled down both harmonin (detected by Ponceau S) and CKK (Fig. 6A: CKK1, Fig. 6B: CKK2, detected by anti-Marshalin), while GST alone did not pull down either harmonin or CKK. To exclude the possibility that bacterial proteins present in the crude lysate mediate the CKK, harmonin, and CDH23-C interactions, we purified CKK1 and harmonin separately using Ni-NTA beads. Excess amounts of purified CKK1 and harmonin were then added to GST-CDH23-C1-bound resin simultaneously at different molar ratios ranging from 1:0 to 1:4 (1 μM of CKK1 to 4 μM of harmonin). The same amount of GST-CDH23-C1 bait, stained by Ponceau S, pulled down both CKK1 and harmonin while GST did not (Fig. 6C, Left). Unbound fractions of CKK1 and harmonin proteins, detected by Coomassie Blue staining, were visible in flow-though (Fig. 6C, Right). Together, these data suggest that CDH23-C isoforms can simultaneously bind to CKK1 and harmonin.

### CDH23-C interferes with MT bundle formation through its N-terminus

**Figure 7:**
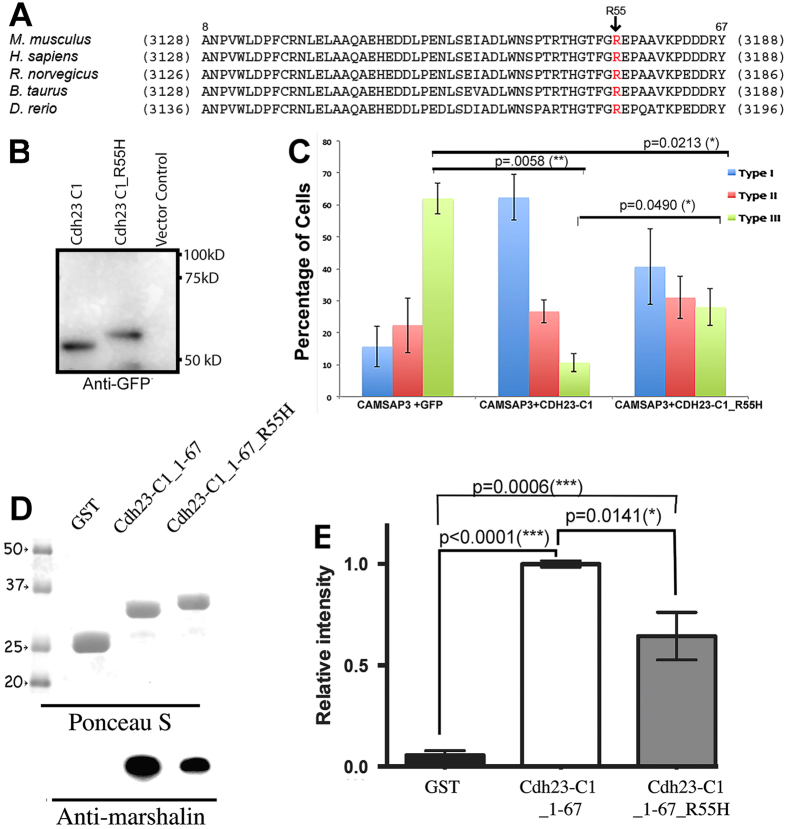
Mutation in the CBM decreases CDH23-C’s ability to bind CKK. (**A)** The R3175 site resides in the CBM, a highly conserved cytoplasmic region of CDH23. Amino acid sequences of CBM from mouse CDH23-A1 (NM023370) are aligned with corresponding sequences from four species (human, NM022124; Rat, BAB61904; Cattle, DAA14294; Zebrafish, AAS98176). Numbers in parentheses indicate residue numbers at the beginning and end of CBM. Residue numbers corresponding to mouse CDH23-C1 are indicated above the mouse CDH23-A1 sequence. Location of mutation R3175H is shown with an arrow. (**B)** Western Blot of CDH23-C1-GFP and CDH23-C1_R55H-GFP, detected by anti-GFP. Change from R to H significantly alters the apparent molecular weight of CDH23-C1_R55H, which is larger than CDH23-C1. (**C**) *In vitro* MT formation assay. A bar graph shows the distribution of three expression patterns (as in Fig. 3A) of CAMSAP3/Marshalin-Ld in OK cells 24 hours post transfection with plasmids encoding *CAMSAP3/Marshalin-Ld* and *Cdh23-C1-GFP* or *Cdh23-C1_R55H-GFP,* respectively. CDH23-C1_R55H reduces type III MT bundle formation to some degree, but less effectively than CDH23-C1 (*p* = 0.0490, n = 3, mean ± s.e.m.). (**D**,**E**) GST pull-down assays of CDH23-C1 fragments. Binding between Cdh23-C aa 1–67 with or without R3175H mutation (CDH23-C_1–67_R55H) and CKK is significantly different. Compared to the negative control (GST), both CDH23-C_1–67 and CDH23-C_1–67_R55H bind to CKK (*p* ≤ 0.0001 and *p* = *0.0006*, respectively). However, the binding to CKK is consistently reduced in CDH23-C_1–67_R55H compared to WT (*p* = 0.0141, n = 4, mean ± s.e.m.). Molecular weights (in kD) are labeled with numbers and arrowheads.

Mutations in *CDH23* cause Usher Syndrome type 1D. One disease-associated missense mutation occurs within the intracellular region that changes arginine to histidine at the amino acid position 3175 of *CDH23-A1* (R3175H)^32^. It is not clear how this particular mutation results in Usher Syndrome type 1D, as CDH23_R3175 is expressed and properly targets the plasma membrane^36^. This mutation is also not associated with any known domain/motif that would directly influence CDH23’s adhesive function or interactions with other protein partners within the cytoplasm. It is intriguing, however, that R3175 resides within the CBM that is involved in the binding to the CKK domain of CAMSAP3/Marshalin (Fig. 5D). Figure 7A shows the alignment of CBM amino acid sequences in five species: human (NM_022124.5), mouse (NM_023370.3), rat (BAB61904.1), cow (DAA14294.1) and zebrafish (AAS98176.1). The sequence corresponds to residues 8–67 of CDH23-C1 as the first 7 amino acids of CDH23-C1 are unique to CDH23-C isoforms due to alternative gene splicing. Among the 60 aa composing the CBM, only 6 differ among the five species ranging from zebrafish to human, i.e., 90% of the amino acids in CBM are identical. R3175 of human CDH23-A1 is equivalent to amino acid 55 within mouse *Cdh23-C1*, which is also conserved among all species. We suspected that mutation of this highly conserved, positively charged amino acid might affect CDH23-C and CAMSAP3/Marshalin-Ld binding. Therefore, we introduced the same mutation within our GST-CDH23-C1 construct at position 55 (CDH23-C1_R55H), which corresponds to the R3175H mutation found in Usher Syndrome type 1D patients. This point mutation altered migration of the CDH23-C1 protein compared to the wildtype in SDS-PAGE (Fig. 7B). The increased apparent molecular mass for CDH23-C1_R55H suggests that CDH23-C1_R55H may be less compact than wildtype CDH23-C1. We also investigated the effects of CDH23-C1_R55H protein on CAMSAP3/Marshalin-Ld-induced MT bundle formation. As shown in Fig. 7C, OK cells co-expressing CAMSAP3/Marshalin-Ld and CDH23-C1-GFP predominantly displayed type I patterns shown in blue (fewer bundles, see Fig. 3A), while the majority of OK cells co-expressing CAMSAP3/Marshalin-Ld and GFP displayed type III patterns shown in green (more bundles, see Fig. 3A). The percentage of cells displaying type III patterns decreased significantly in the presence of CDH23-C1 (p < 0.01). Interestingly, OK cells co-expressing CDH23-C1_R55H-GFP showed bundle phenotypes with no dominant pattern. This observation suggests that CDH23-C1_R55H protein inhibits CAMSAP3/Marshalin-induced MT bundle formation as the percentage of cells displaying type III patterns significantly decreased in the presence of CDH23-C1_R55H (*p* < 0.05) as compared to GFP controls, albeit less effectively than wildtype CDH23-C1. In other words, more cells showed the type III phenotype in OK cells co-expressing CAMSAP3/Marshalin-Ld and CDH23-C1_R55H-GFP than OK cells co-expressing CAMSAP3/Marshalin-Ld and wildtype CDH23-C1-GFP (*p* < 0.05). Since the R55H mutation is within the CBM of CDH23-C isoform, we also introduced R55H into the CDH23-C1_1–67 construct, and performed the GST pull-down assay. The resulting GST-CDH23-C1_1–67_R55H fragment also exhibited a slight increase in the apparent molecular weight on the gel compared to the WT (Fig. 7D, top). Consistent with the MT bundle formation assay, GST-CDH23-C1_1–67_R55H showed a statistically significant reduction in CKK binding compared to the wildtype (Fig. 7D, bottom, and Fig. 7E, *p* = 0.0141). These data suggest that the mutation within the CBM of CDH23-C isoforms alters the interaction between CDH23-C and CAMSAP3/Marshalin-Ld.

## Discussion

CDH23 is an essential protein for auditory and visual signal transduction and is important for cell adhesion. Function of the cytoplasmic isoforms, however, is not fully understood even though CDH23-C is detected in various tissues and implicated in cochlear hair cell development^13^. In this study, we demonstrated that CAMSAP3/Marshalin is a new CDH23-associated protein. Although the interaction between these two proteins was initially found using yeast two-hybridization screening^20^, their interaction was verified in this study through a combination of *in vivo* MT bundle formation assays within mammalian cells and *in vitro* GST pull-down assays. We have identified the binding sites between CDH23-C and CAMSAP3/Marshalin-Ld in the N-terminus of CDH23-C (CBM) and the CKK domain of CAMSAP3/Marshalin-Ld, respectively. We further showed that mutation in the CMB of CDH23, which was discovered in an Usher Syndrome 1D patient (R3175H), resulted in a marked decrease in CDH23-C’s ability to interact with CAMSAP3/Marshalin both *in vivo* and *in vitro*. These data suggest that CDH23-C plays an important role in regulating MT networks via its interaction with CAMSAP3/Marshalin, and that alteration of this interaction can lead to pathological conditions such as Usher Syndrome.

As an adhesive protein, CDH23 is generally thought to localize to the cell surface. Therefore, it was puzzling when CDH23 was also found in MT-rich areas such as centrosomes and basal bodies that are internal structures^10^,^12^. Unlike previously described CDH23-associated proteins, CAMSAP3/Marshalin-Ld is a rare MT minus-end-binding protein that directly associates with MTs. A direct connection between CDH23-C and CAMSAP3/Marshalin-Ld may explain the mysterious staining of CDH23 in MT-rich areas, and immediately suggests that CDH23-C plays an important role in regulation of MT networks within hair cells where both CDH23 and CAMSAP3/Marshalin are co-expressed. This intracellular staining may be especially important for synaptic development^13^,^21^. Since all CDH23 isoforms have CBM, CDH23-A and CDH23-B isoforms could tentatively interact with CAMSAP3/Marshalin-Ld to organize MT networks. Since CAMSAP3/Marshalin is also commonly expressed in various cell types, it is reasonable to assume that CDH23 serves to regulate MT networks in other tissues besides those related to hearing and vision.

**Figure 8:**
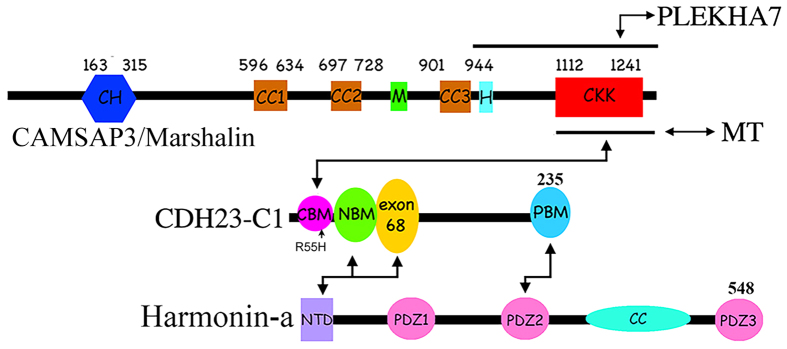
A schematic representation of protein-protein interactions connecting CDH23-C1, CAMSAP3/Marshalin, and harmonin-a. The connection between CDH23-C1 and CAMSAP3/Marshalin influences MT organization, and the mutation within CBM (R55H), which affects the interaction between CDH23-C and CAMSAP3/Marshalin is found in Usher Syndrome 1D patients.

CDH23 is known to bind to the PDZ domain of harmonin through multiple domains including PBM, exon 68, and NBM^18^,^19^. This connection links CDH23 with other proteins that may influence the hair cell transducer apparatus that converts sound waves into electrical signals. CDH23 can also interact with proteins without a PDZ domain such as the transport protein EHD4^15^. In this study, we identified a new protein-protein interaction domain, CBM, at the very beginning of the N-terminus of CDH23-C (Fig. 8). As the smallest isoform of CDH23, which is not tethered to the plasma membrane, CDH23-C has more freedom of movement than its larger isoforms allowing it to interact with associated proteins such as harmonin and CAMSAP3/Marshalin. Both harmonin and CAMSAP3/Marshalin have multiple isoforms that carry several protein-protein interaction domains. Our data show that binding of CKK to CDH23-C can occur in the presence harmonin and more importantly does not prevent harmonin/Cdh23-C interactions. The latter are required for CDH23’s interaction with actin-based microfilaments^37^. The CKK domain of CAMSAP3/Marshalin is also known to interact with MTs and PLEKHA7, in addition to CDH23-C (Fig. 8). Whether CDH23-C interferes with the binding between PLEKHA7 and CKK requires investigation.

Our data suggest that CDH23-C can regulate MT networks indirectly through its interaction with CAMSAP3/Marshalin. As a new modifier of MT networks, additional studies are required to further define CDH23-C’s roles in various physiological contexts that may ultimately impact our understanding of the fundamental mechanisms associated with hearing and vision.

## Materials and Methods

### DNA constructs

For mammalian expression, the cDNA of mouse *CAMSAP3/Marshalin-Ld* or the CKK domain (CKK1, residues 1020–1253) was inserted into a pcDNA6/V5His vector to introduce a V5-His tag to the C-terminus^21^. *GFP-Cdh23-C1* and *GFP-Cdh23-C2* constructs were kindly provided by Dr. Friedman^10^. A point mutation in CDH23-C isoforms (R55H, corresponding to CDH23 mutant R3175H in humans)^32^ was introduced into *GFP-Cdh23-C1* using a QuickChange Site-Directed Mutagenesis Kit (Stratagene) to generate *GFP-Cdh23-C1_R55H*. For bacterial expression, CKK domains of mouse *CAMSAP3/Marshalin-Ld* (CKK1, residues 1020–1253; CKK2, residues 1112–1253) were inserted into the NheI/SalI site of the pET24(a) vector. Both constructs contained a His-tag at the C-terminus. We also made a construct encoding CKK1 cDNA without the His-tag. Glutathione S-Transferase (GST)-tagged mouse CDH23-A cytoplasmic tail constructs, CDH23, CDH23 (+68), CDH23 (+68, Δ4), CDH23 (Δ108), and CDH23 (Δ143), were generous gifts from Dr. Bartles^19^. The numbers after Δ are the residue numbers eliminated from the C-terminus of CDH23. Various *Cdh23-C1* and *Cdh23-C2* fragments were inserted into the EcoRI/XhoI site of the pGEX-4T-2 vector with an N-terminal GST tag to generate the following constructs: *Cdh23-C1*, *Cdh23-C2*, *Cdh23-C_1–67* (first 67 aa of CDH23-C), *CDH23-C_1–67_R55H* (first 67 aa of CDH23-C with the R55H mutation).

### Antibodies

Anti-Marshalin^21^ was used at 2 μg/ml for immunofluorescence (IF) and 0.6 μg/ml for Western blot. Commercial antibody dilutions were as follows: anti-V5 (Invitrogen), 1:1,000 dilution for IF and 1:5,000 for Western blot; anti-α-tubulin (Zymed, San Francisco, CA), 1:800 for IF; anti-GFP (Clontech, Mountain View, CA), 1:2,000 for Western blot; anti-GST (Sigma), 1:10,000 for Western blot; goat anti-mouse IgG-Alexa Fluor 546, goat anti-rabbit IgG-Alexa Fluor 488 or 546 (Molecular Probes, Eugene, OR), 1:500 for IF; goat anti-rabbit IgG-HRP and goat anti-mouse IgG-HRP (Jackson ImmunoResearch, West Grove, PA), 1:10,000 for Western blot; Texas Red-X phalloidin (Molecular Probes, Eugene, OR), 1:2,000 to stain F-actin. Donkey anti-mouse IgG-Alexa Fluor 647F(ab’)_2_ Fragment (Jackson ImmunoResearch, West Grove, PA), 1:1,000 for IF.

### *In vitro* GST pull-down assay

Expression of GST-tagged CDH23 cytoplasmic tail proteins or various *Cdh23-C1* and *Cdh23-C2* fragments, His-tagged CKK1/2 and harmonin were induced by 1 mM Isopropyl β-D-1-thiogalactopyranoside (IPTG, Sigma) in *E. coli* BL21 (DE3) cells (Invitrogen). Cells were then lysed in PBS containing 1X protease inhibitor (Sigma), 1 mM phenylmethanesulfonylfluoride (PMSF, Sigma), 1 mg/ml lysozyme (Sigma), and 370 unit/ml Benzonase (Sigma) by sonication. The bacterial lysates were incubated at 4 °C for 30 minutes, then centrifuged at 14000 rpm for 30 minutes at 4 °C to separate insoluble fragments from supernatant. GST-tagged Cdh23 tail proteins and various Cdh23-C1 and Cdh23-C2 fragments were then immobilized on glutathione-agarose (Thermo) at 4 °C on a rotator for 1.5 hours. Unbound proteins were washed away with chilled PBS. For Cdh23- CAMSAP3/Marshalin pull-downs, Cdh23 bound glutathione-agarose (Cdh23-GST-agarose) was incubated with CKK lysate at 4 °C on a rotator for 1.5 hours or overnight. In some experiments, CKK proteins were purified first using a MagneHis Protein Purification System (Promega) before adding 30–60 ng/ml of CKK protein to the Cdh23-GST-agarose. For pull-downs including harmonin, uninduced harmonin bacterial lysate (no harmonin expression) and induced harmonin bacterial lysate were combined with CKK lysate in a 1:1 ratio, and added the Cdh23-GST-agarose. Following incubation, the resins were washed with chilled PBS, and bound proteins were eluted with 100 mM L-Glutathione (Sigma), and then loaded onto 12% (w/v) polyacrylamide-SDS gels. In some experiments, CKK proteins and harmonin were purified first using a MagneHis Protein Purification System. The imidazole in elution buffer was removed and replaced with PBS using PD MiniTrap G25 (GE Healthcare). Protein concentrations were measured used a Pierce 600 nm Protein Assay Reagent (Thermo Scientific). Appropriate molar concentrations of CKK1 and harmonin were then added to the Cdh23-C1-GST-agarose. To visualize enriched GST-tagged proteins/fragments, Coomassie Blue (Thermo) was used to stain the gels, or 5% Ponceau S (Sigma) to stain the nitrocellulose membranes. To probe for CKK domains, membranes were incubated with anti-Marshalin followed by HRP-conjugated secondary antibodies and a SuperSignal West Pico Chemiluminescent Substrate (Thermo). Images were taken with a Kodak Imaging System and the intensities of the expected CDH23 and CKK bands measured using Molecular Imaging software from Kodak (Rochester, NY). For statistical analysis, the signal intensities of CKK were normalized to the intensities of Cdh23-GST bait, and means from 4 independent experiments were plotted. Significance was determined using one-way ANOVA with the Tukey’s multiple comparisons test in Prism 6 (GraphPad).

### MT bundle formation assay

Plasmids encoding V5-His tagged CAMSAP3/Marsalin-Ld or simply its CKK domain were transiently co-transfected with GFP, CDH23-C1-GFP, and CDH23-C2-GFP into opossum kidney (OK) cells as previously described^38^. OK cells were obtained from ATCC (catalog No. CRL-1840) and cultured in MEM medium supplied with 10% fetal bovine serum, 100 unit/ml penicillin, and 100 μg/ml streptomycin in 5% CO_2_ in air at 37 °C. 24–45 hours post transfection, cells were treated with colchicine at a concentration of 250 ng/ml for 24 hrs or 1 μg/ml for 3 hrs. We also utilized LLC–PK1–CL4 cells (CL4 cells) cloned from LLC–PK1 cells provided by Dr. Bartles (Northwestern University). Detailed culture conditions for these cells were described in a previous publication^19^. Both chemically treated and untreated cells were fixed with 2% formaldehyde in PBS for 10 minutes, and incubated with monoclonal anti-V5, anti-Marshalin, or anti-α-tubulin antibodies for 1 hour at room temperature followed by appropriate secondary antibodies (goat anti-mouse IgG-Alexa Fluor 546 or goat anti-rabbit IgG-Alexa Fluor 488) for 1 hour as previously described^21^. For each sample, 100–200 cells were randomly selected for analyzing CAMSAP3/Marshalin-Ld expression patterns using an epifluorescence microscope (Micros, Austria). Images were captured on a Nikon C2+ or A1R confocal microscope equipped with laser lines including 408, 458, 488, 515, 561, 638 nm, and with Plan Apo 20X objectives (Nikon) and a Plan Apo 60X oil objective (Nikon) controlled by NIS Element software. Significance was determined using the two-tailed Student’s t-test.

### Western blot analysis

OK cells transiently transfected with *GFP-Cdh23-C1* or *GFP-Cdh23-C1_R55H* were harvested 2 days after transfection and lysed in cold lysis buffer (50 mM Tris-HCl, pH 7.6, 150 mM NaCl, 1% Triton X-100) supplemented with 1X protease inhibitor cocktail and 1 mM PMSF (Sigma). Insoluble material was removed by centrifugation at 15,000 g for 15 minutes at 4 °C. Proteins were resolved using 8% SDS-PAGE, followed by immunoblotting using anti-GFP (Rockland). Signals were detected using a SuperSignal West Pico Chemiluminescent Substrate (Thermo). A Kodak Imaging System was used to capture the images.

## Additional Information

**How to cite this article**: Takahashi, S. *et al*. Cadherin 23-C Regulates Microtubule Networks by Modifying CAMSAP3’s Function. *Sci. Rep.* **6**, 28706; doi: 10.1038/srep28706 (2016).

## Supplementary Material

Supplementary Information
