# Supplementary material for: Cadherin 23-C Regulates Microtubule Networks by Modifying CAMSAP3’s Function
Source: Sci Rep. 2016 Jun 28;6:28706. doi: 10.1038/srep28706 (PMC4923861; doi:10.1038/srep28706)
Supplement: Supplementary Information [file srep28706-s1.pdf]

Supplementary Information

Cadherin 23-C Regulates Microtubule Networks by Modifying CAMSAP3's Function

Satoe Takahashi[1], Vincent J. Mui[1], Samuel K. Rosenberg[1], Kazuaki Homma[1, 3], Mary Ann Cheatham[2, 3], and Jing Zheng[1, 3#]

[1]Department of Otolaryngology – Head and Neck Surgery, Feinberg School of Medicine, Northwestern University, Chicago IL 60611
[2]Department of Communication Sciences and Disorders, Northwestern University, Evanston, IL 60208
[3]Knowles Hearing Center, Northwestern University, Evanston, IL 60208

#Corresponding author:
Jing Zheng
Department of Otolaryngology – Head and Neck Surgery
Feinberg School of Medicine, Northwestern University
320 East Superior Street
Chicago, IL 60611
jzh215@northwestern.edu
Tel: (312)-503-3417.
Fax: 412-503-1616

Figure S1

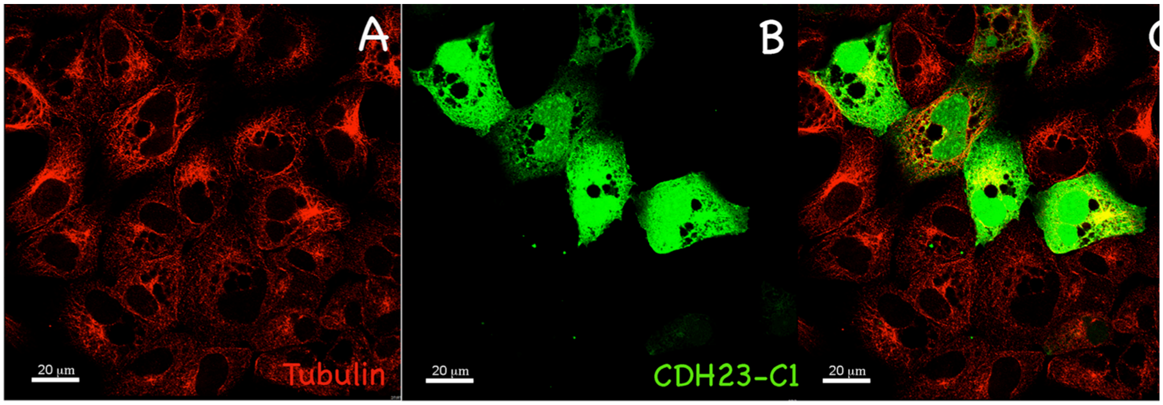

Fig. S1. Immunofluorescent images showing MT networks in OK cells transfected with *Cdh23-C1-GFP*. Note that CDH23-C1-GFP does not induce MT-bundles in the absence of CAMSAP3/Marshalin-Ld, as MT morphology in cells expressing CDH23-C1-GFP (green) is similar to that in cells without CDH23-C1-GFP. Red: anti-α-tubulin.

Figure S2

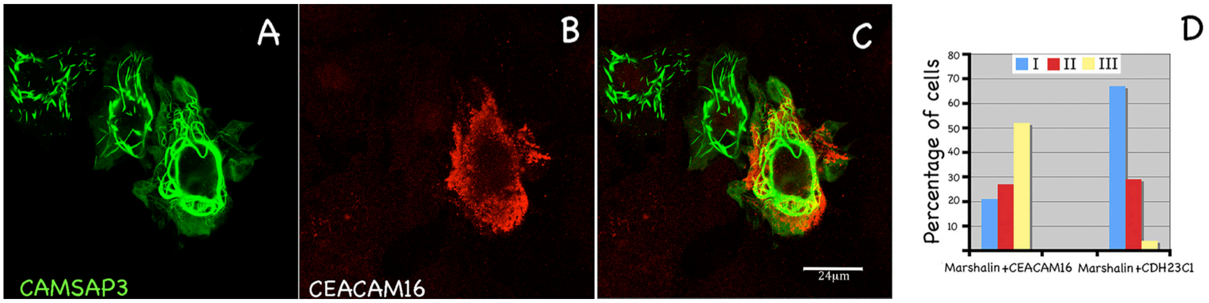

Fig. S2. Ceacam16 protein does not interfere with CAMSAP3/Marshalin-Ld induced MT bundle formation. **A-C.** Immunofluorescent images show MT bundles induced by CAMSAP3/Marshalin-Ld. A merged image (**C**) shows both CAMSAP3/Marshalin-Ld (green) (**A**) and Ceacam16 (red) (**B**). **D.** A bar graph shows the distribution of three expression patterns (as in **Fig. 3A**) of CAMSAP3/Marshalin-Ld in OK cells 48 hours after co-transfection with CAMSAP3/Marshalin and CEACAM16 (left), or CAMSAP3/Marshalin and CDH23-C1 (right).

Figure S3

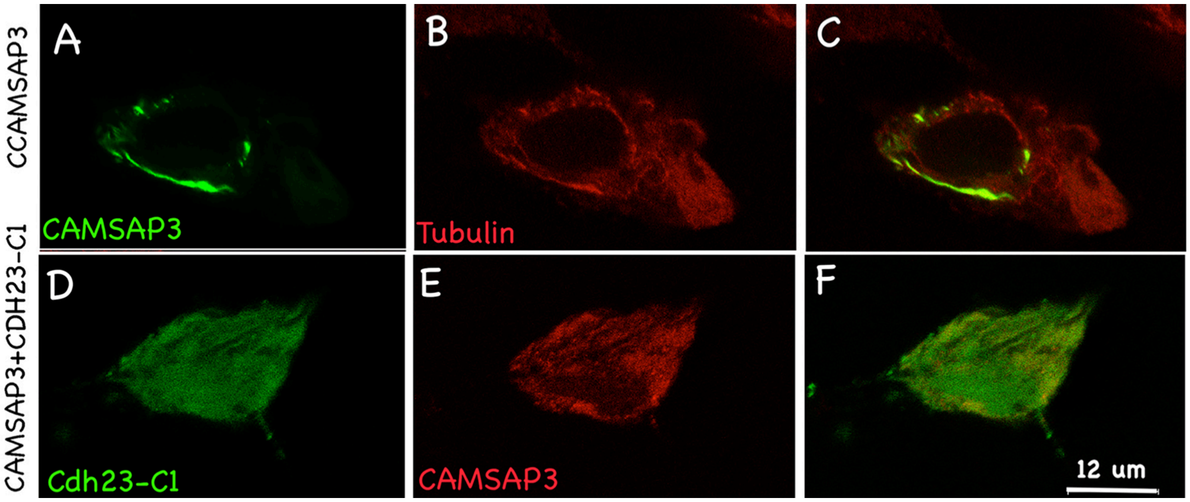

Fig. S3. CAMSAP3/Marshalin-Ld in LLC-PK1-Cl4 cells. **A-C.** LLC-PK1-Cl4 cells transfected with *Camsap3/Marshalin-Ld* alone. A merged immunofluorescent image (**C**) shows tubulin (**B**, red) and CAMSAP3/Marshalin-Ld (**A**, green). **D-F.** A merged immunofluorescent image (**F**) shows CAMSAP3/Marshalin-Ld (**E**, red) and CDH23-C1 (**D**, green) staining in LLC-PK1-Cl4 cells transfected with plasmids encoding CAMSAP3/Marshalin-Ld and CDH23-C1.

Figure S4.

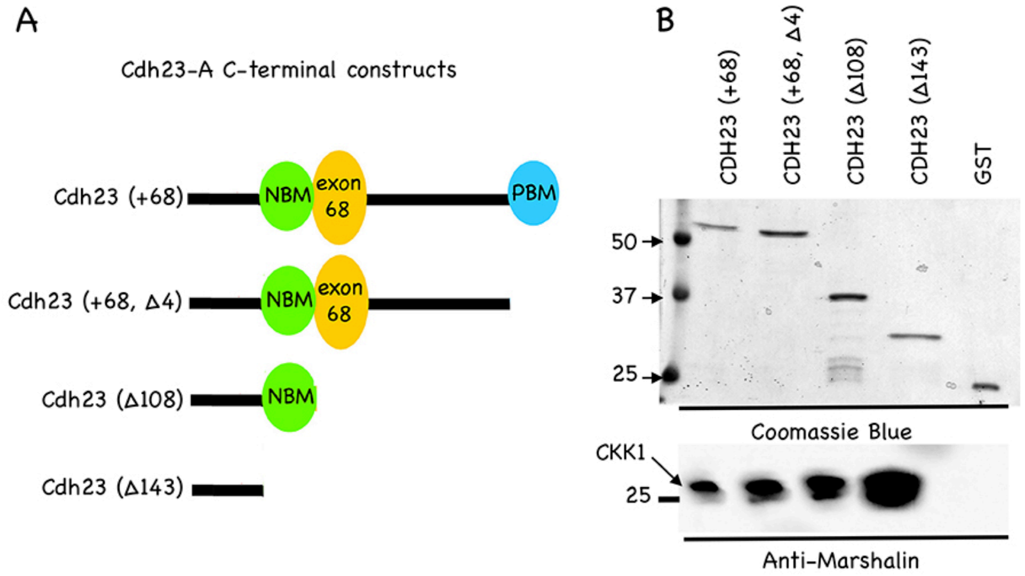

Fig. S4. Harmonin interaction domains of CDH23 PBM, exon 68, and NBM are not required for the CKK binding. **A.** List of CDH23-A cytoplasmic domain constructs containing deletions from the C-terminus. Cdh23 (+68), cytoplasmic tails from CDH23-A1 and A2, differ from the C isoforms by having an extra 62 amino acids ahead of the N-terminus of the CDH23-C isoform and by lacking 7 residues unique to C isoforms. Cdh23 (+68, Δ4) lacks the C-terminal PBM, Cdh23 (Δ108) lacks exon 68, and Cdh23 (Δ143) lacks all three. **B.** GST pull-down assay showing interaction between CDH23-A cytoplasmic fragments and the CKK domain. GST, and GST-tagged C-terminal fragments of CDH23-A are immobilized on glutathione agarose, and incubated with cell lysates containing CKK1 proteins. GST-fusion proteins were visualized by Coomassie Blue (top); CKK1 proteins with anti-Marshalin.
